# Evaluation of Several Feature Detectors/Extractors on Underwater Images towards vSLAM

**DOI:** 10.3390/s20154343

**Published:** 2020-08-04

**Authors:** Franco Hidalgo, Thomas Bräunl

**Affiliations:** 1 Facultad de Ingeniería, Universidad San Ignacio de Loyola, La Molina, Lima 15024, Peru; 2 Department of Electrical, Electronic and Computer Engineering, The University of Western Australia, Perth, WA 6009, Australia; thomas.braunl@uwa.edu.au

**Keywords:** vSLAM, detector, descriptor, underwater video, monocular underwater, underwater robots, SIFT, SURF, ORB, AKAZE, BRISK

## Abstract

Modern visual SLAM (vSLAM) algorithms take advantage of computer vision developments in image processing and in interest point detectors to create maps and trajectories from camera images. Different feature detectors and extractors have been evaluated for this purpose in air and ground environments, but not extensively for underwater scenarios. In this paper (I) we characterize underwater images where light and suspended particles alter considerably the images captured, (II) evaluate the performance of common interest points detectors and descriptors in a variety of underwater scenes and conditions towards vSLAM in terms of the number of features matched in subsequent video frames, the precision of the descriptors and the processing time. This research justifies the usage of feature detectors in vSLAM for underwater scenarios and present its challenges and limitations.

## 1. Introduction

Knowing the position of underwater robots and obtaining maps of the surrounding environment is essential for a variety of robot tasks, from gathering geo-referenced data to autonomous navigation and exploration. Simultaneous Localization and Mapping (SLAM) offers a framework to incrementally build a map while a robot moves through an unknown area and to use that map to localize the robot simultaneously. A typical implementation of SLAM in the underwater environment involves the use of dead-reckoning, acoustic sensors and cameras [1]. In the last few years, the use of cameras as the primary sensor for SLAM has increased. This branch of SLAM is also referred to as visual SLAM (vSLAM) which mainly focuses on estimating the pose of the camera from partially overlapping images from different viewpoints and creates a map of images or a cloud of points. Visual SLAM can be categorized based on how the images are processed in direct algorithms, where complete image intensities are processed, and feature-based, where only certain key-points of the image are computed [2].

A fundamental part of feature-based SLAM is data association which allows extracted features from images as key-points to be recognized when re-observed in consecutive images as well as in loop-closing. To achieve this, features are extracted using an interest point detector, and then described including local information from the neighbors of the point through a feature descriptor. The descriptor is a vector which assigns a distinctive identity to the feature to be recognizable [3].

In computer vision there are several feature detectors and descriptors which have been evaluated in terms of scale invariance, viewpoint changes (including rotation) and variations in illumination [4,5], as well as their application to vSLAM [3,6,7]. There are successful implementations of vSLAM for underwater robots such as in [8,9,10] which relies on Speeded-Up Robust Features (SURF), Scale Invariant Feature Transform (SIFT) feature detectors and other methods to extract regions of interests. To the best of the authors’ knowledge, there is not extensive documentation which analyses feature detectors and descriptors for underwater environments. This might be related to the higher number of applications of point detectors and descriptors in indoors environments and, in images captured by ground or air robots compared to underwater environments which present images with dynamic illumination, blurriness, turbidity; and there are fewer targets from which features can be extracted, mostly limited to man-made structures, animals or the seafloor, which can be affected by the currents such as in the cases of sand patches and algae.

Underwater images are subject to alterations to the light and characteristics of the medium resulting in blurry, hazy and tinted images [11]. This presents challenges to the performance of feature detectors towards vSLAM. Therefore, we propose a characterization of underwater scenarios based on a variety of datasets in different conditions and include some processed datasets through enhancing algorithms. We evaluate the response and performance of common feature detectors and descriptors such as SIFT, SURF, Oriented FAST and Rotated BRIEF (ORB), Binary Robust Invariant Scalable Keypoints (BRISK) and AKAZE in matching consecutive images towards its application in vSLAM. Finally, we compare the computation time for the features detection and matching.

In the second part of this Section, a selection of related works to feature detectors and their evaluation in vSLAM is presented. Section 2 presents a brief overview of selected feature detectors with their corresponding descriptor. Section 3 presents the alterations found in underwater images as well as a brief description of enhancement algorithms. The evaluation methodology and the results are presented in Section 4 and Section 5. Finally, the results are discussed in Section 6 together with the conclusions.

### 1.1. Related Work

**Figure 1:**
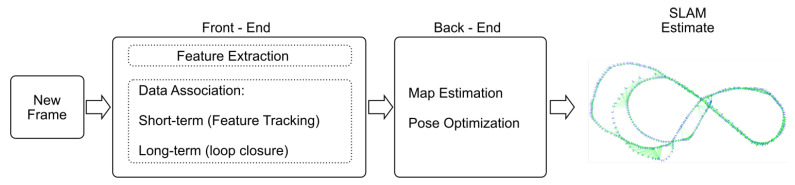
Simplified vSLAM architecture.

In [2], Younes et al. presented a survey on monocular visual SLAM outlining a general guideline of a monocular keypoints SLAM system in which seven components were defined: “visual initialization, data association, pose optimization, topological/metric map generation (map expansion), bundle adjustment/pose-graph optimization/map maintenance, failure recovery and loop closure”. In [12], typical modern SLAM architectures are abstracted to front-end and back-end components. The first one extracts sensors data and pre-processes it to be handled by the back-end to infer a consistent map and pose estimation. In this representation, the data association process fits in the front-end leaving the other modules to the back-end Figure 1.

For feature-based vSLAM, the front-end involves the detection of interest points, the creation of descriptors, and the data association performed by matching features from the current frame with previous frames.

### 1.2. Feature Detectors in Visual SLAM

Visual SLAM approaches have been evaluated for indoor and outdoor applications over benchmark datasets. In [13] ORB-SLAM, Large Scale Direct SLAM (LSD-SLAM), Low dimensionality SLAM (L-SLAM) and open source of RatSLAM algorithms are briefly described and assessed. ORB-SLAM shows good results for different environments presenting the smallest errors when compared to LSD-SLAM and Rat-SLAM. The authors also pointed out the need of manual post-processing to reduce the error since the maps and trajectories need post-scaling to fit proper dimensions.

In [14] an experimental evaluation of the algorithms was performed for different datasets collected on land, aerial and underwater vehicles. They found, again, a good performance by ORB-SLAM and Parallel Tracking and Mapping (PTAM) for the majority of scenarios. Finally, another evaluation was performed in [7] having similar results with three different feature detectors: Harris, Kanade-Lucas Tracker (KLT) and SIFT.

### 1.3. Feature Detectors Evaluation

Several feature detectors and descriptors have been evaluated in the past regarding correct matching against image alterations. For this purpose, features are extracted from a pair of images from the same scene or digitally altered and then matched against the other. In [15] the SIFT descriptor was evaluated with ground truth showing robustness against rotation, scale, viewpoint changes, image blur and light change. They define a few ratios, first used in [16], to measure the performance of the measurements.

(1) $$recall=\frac{\#correct\_matches}{\#correspondences}$$

The recall ratio Equation (1) equivalent to the detection rate where #correct_matches are the features matched correctly in both images.

(2) $$1-precision=\frac{\#false\_matches}{\#matches}$$

The 1−precision ration Equation (2) which indicates the relative number of false matches, where #false_matches is the number of matches that do not correspond features found in both images and #matches is the total number of matches.

Johansson et al. used the same performance ratios to evaluate more detectors and descriptors; and combinations (detectors/descriptors). They include SURF, ORB, BRISK, Fast Retina Keypoint (FREAK) finding the combination SURF/SURF and ORB/BRISK robust against geometric and photometric transformations [17]. Similarly, Gil et al. evluated SURF and Gradient Location and Orientation Histogram (GLOH) (a SIFT like descriptor) suitable for a vSLAM application [3].

Other evaluations are carried out for customized functions such as for tracking objects [18] and vision-based localization [19]. In [19] they added the Accelerated-KAZE (AKAZE) detector/descriptor to the review and analyzed the computing time. They also included Compute Unified Device Architecture (CUDA) implementations of AKAZE and SIFT being the fastest two in extracting, detecting and matching, followed by ORB and SURF. SIFT appeared as the slowest followed by AKAZE and BRISK. Additionally, they added repeatability, precision and accuracy as comparison criteria.

## 2. Selected Feature Detectors and Descriptors

Based on the performance of features extractors in the literature discussed in Section 1 we select SIFT [20], SURF [21], ORB [22], AKAZE [23] and BRISK [24] which are robust and have been used for indoor and outdoor environments in [19,25]. In Table 1 the characteristics of the detectors and descriptors are presented, as well as some parameters based on their OpenCV implementation.

### 2.1. Scale Invariant Feature Transform (SIFT)

The SIFT algorithm follows two main stages in the detection part: (1) Scale-space extrema detection, where Difference of Gaussian (DoG) is applied to identify keypoint invariants to scale changes, then a local extrema check with adjacent pixels is performed; (2) keypoint localization, which rejects low contrast keypoints and then eliminates non-edge points based on Hessian matrix.

For building the descriptor the algorithm follows two further steps: (3) orientation assignment, which forms orientation histograms from local gradients to determine the dominant direction of the keypoint; (4) keypoint descriptor, where the proper vector is constructed based on the course of the keypoints and local areas around them, and finally the descriptors are normalized to improve light invariance [20,26].

### 2.2. Speeded-Up Robust Features (SURF)

SURF follows a similar idea as SIFT, it was developed by Bay et al. [21] as a faster and robust alternative to previous extractors. It uses integral images [27] and simplified filter kernels compared to SIFT through a Fast-Hessian detector based on 2D Haar wavelet response.

The descriptor combines local gradient information, like SIFT, 2D Haar wavelet response to local areas and windows around they keypoints to approximate the gradients.

### 2.3. Oriented FAST and Rotated BRIEF (ORB)

ORB is based on Features from Accelerated Segment Test (FAST) and Rotated BRIEF. It creates a pyramid of blurred and subsample versions of the image which are then divided into cells and FAST is computed. Then the cells are subdivided to contain one corner per cell or the maximum number of cells allowed by a parameter of the algorithm, disregarding the features with low score per cell.

The ORB descriptor modifies the FAST extractor adding an orientation component through first-order moments in a local patch. Then the Binary Robust Independent Elementary Features (BRIEF) descriptor is computed on a rotated patch. It reduces the descriptor vectors such as in SIFT and SURF to binary vectors [22].

### 2.4. Binary Robust Invariant Scalable Keypoints (BRISK)

BRISK is based on the FAST detector, it extracts features from the image and different scales of it. For the descriptor, it uses a concentric rings sampling pattern to retrieve the gray values of their neighbors and process local intensity gradients to obtain the direction of the keypoint. Then it forms the binary descriptor comparing the intensity between pairs from the pattern [24].

### 2.5. Accelerated-KAZE (AKAZE)

AKAZE focuses on multi-scale feature detection exploiting non-linear scale spaces. It is computationally efficient taking advantage of Fast Explicit Diffusion. It applies the Hessian determinant to the scaled images and performs a search of the maxima response in spatial location.

Alcantarilla et al. proposed a Modified-Local Difference Binary (M-LDB), that exploits gradient and intensity from the extractor stage, as a descriptor. It is based on BRIEF performing over the average of areas instead of pixels. It includes intensity values, and the orientation of the keypoint is similar to KAZE [23].

## 3. Underwater Monocular Images

**Figure 2:**
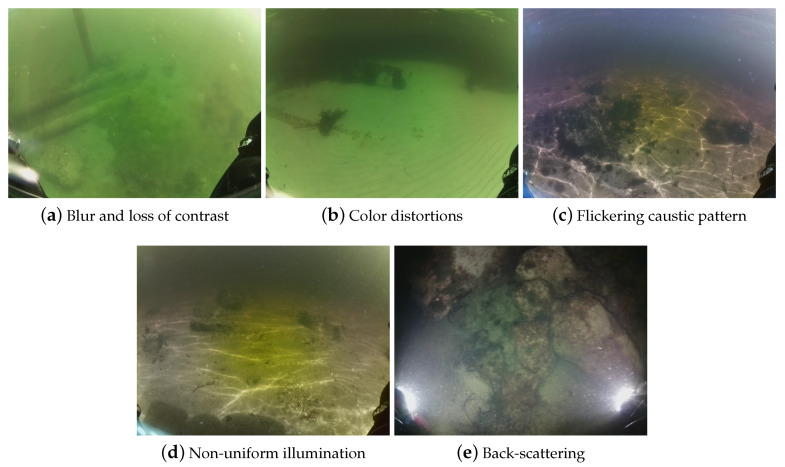
Lighting effects on underwater images.

Images captured in underwater scenarios are altered in every aspect due to the changes in radiant energy when traveling through water rather than air. Light gets scattered by suspended tiny particles in the water (quartz sand, clay mineral, plankton, etc.) and it is also absorbed by the water itself causing blur and loss of contrast (Figure 2a) [28]. The energy absorption varies with wavelengths and types of water (i.e. sea, fresh and variations in its composition), generating perceived color distortions (Figure 2b) at different distances and types of water. Additionally, changes in perception of size and distance also occur in underwater scenarios and are caused by the light refraction as it passes from air to water [11].

Sunlight flickers (caustic waves) are observed in very shallow water which are formed by trespassing a wavy water layer [29]. These lighting variations generate flickering caustic patterns (Figure 2c), which can be seen as random thin bright traces and non-uniform illumination, which are observable as brighter small patches (Figure 2d) [8].

Artificial light sources are used when gathering images at night or in murky water to increase the lightness of the scene. The source is usually located near the camera and the light is reflected by particles in the medium yielding the back-scatter component (Figure 2e) [30,31].

### Underwater Image Enhancement

There are several approaches of image processing to enhance underwater images regarding the lighting effects presented before. In [31], Wang lists around 25 different algorithms for underwater image enhancement and restoration. The author organized them in four categories, having ‘Histogram and Contrast Ratio’, which mainly enhances the contrast; ‘Retinex Model’, with good results in low contrast and non-uniform illumination; ‘Filtering and Transformations’, which also enhances non-uniform illuminated images, corrects the image tone, reduces noise of bright spots and improves contrast; and ‘Comprehensive’, which enhances and restores colors in the images.

Other methods developed mainly to diminish the effect of sunlight flickering such as the works presented in [29,32,33,34]. Additionally, the algorithms presented in [30,35] enhance underwater images with respect to the back-scattering problem. ‘Dehaze’ algorithms have also been used to overcome the light scattering problem in air [36,37,38] and in water [39]. In [40], a method for enhancing images against low contrast and color distortion based on guided filer and color space conversion is introduced.

External hardware have been used for mitigating the lighting problems when gathering underwater images. Treibitz et al. placed polarizers on the light source and the camera to achieve back-scatter reduction [41,42]. In [43], a barrier filter was used in front of the camera for the same purpose.

## 4. Evaluation Framework

In this Section we present the evaluation framework followed, based on the literature described in Section 1. A quantitative and qualitative analysis is performed to evaluate the performance of feature detectors and descriptors applied to underwater images toward their application to vSLAM.

Three key elements to apply the detector/descriptor sets described to a feature-based SLAM are: the capacity to extract keypoints from an image, the capacity to associate re-observed keypoints, and the computational time. In this regards the detectable features and frame sequence matching tests are proposed. Additionally, we include pre-processed images from the datasets through an enhancing underwater image by fusion [44] and backscatter removal to enhance the visibility of underwater objects [35].

We use two detectors’ profile tuned manually to expose features proportionally to a limit. One is set to obtain around 1000 features ($Profile_{1k}$) and the other to achieve a higher value, limited to 10,000 features ($Profile_{10k}$). The profiles are based on the threshold of the extractors and the number of maximum features (Table 2). The other parameters are left to the default values of the OpenCV implementation of the algorithms.

### 4.1. Detectable Features in Underwater Images

We describe a selected number of underwater datasets based on the challenges presented in Section 3 and evaluate different feature detectors on them to determine distinctive image features in underwater scenarios. The features are obtained by applying the OpenCV implementation of the feature extractors to each of the frames of the datasets. Enhanced datasets are also included to examine how the pre-process performs when the feature extractors are applied.

The two features extractors profiles are included in the analysis. Quantitatively, the number of features extracted is given. A qualitative description of the detected features in different scenario conditions is also provided.

### 4.2. Frame Sequence Matching

We evaluate matches between consecutive scenes which are analyzed towards the application of the detectors/descriptors set in vSLAM. This provides insights of the data association process in the location of features from different viewpoints [45,46].

**Figure 3:**
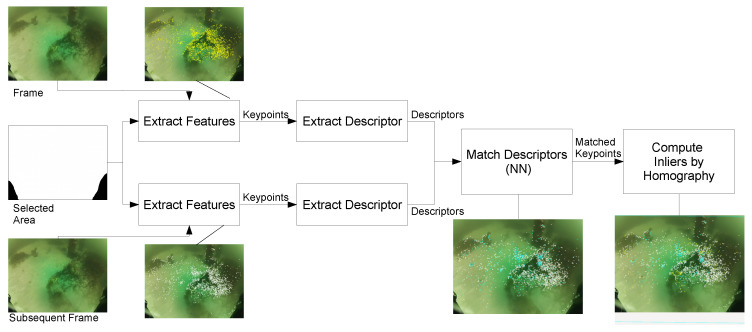
Block diagram of data extraction for evaluation. First, features are extracted and described from two consecutive frames (features in yellow and white for frame and subsequent frame respectively). Then features are matched between both frames (cyan points on the subsequent frame show matches, white points, rest of features). Finally inliers (cyan points) and outliers (yellow points) are computed.

We use a similar approach to the works reviewed in Section 1.3, but since the datasets extract features for real underwater surveys, ground truth of the keypoints was not gathered. Under vSLAM method of connecting frames and features we assume that all the features detected in a frame should appear in the subsequent frame. Additionally, we use the computed number of inliers (correct match of keypoint in both images) and outliers (false match) by homography presented in Figure 3.

We adapt Equation (1) to Equation (3) where #correct_matches becomes #inliers and #correspondences becomes #features. Instead of evaluating Equation (2) we evaluate Equation (4), where precision_mod is the relative number of correct matches obtained from the inliers and outliers. In other words recall_mod gives an idea of the number of good matches it would get in the subsequent frame from the number of features found in the current frame; and precision_mod provides information on the performance of the detector/descriptor worked, from the total matches found, how many are correct.

(3) $$recall\_mod=\frac{\#inliers}{\#features}$$

(4) $$precision\_mod=\frac{\#inliers}{\#matches}$$

### 4.3. Datasets

We collected different datasets for a variety of underwater scenarios in rivers, beaches, ports and open sea in the surroundings of Perth, Australia (http://robotics.ee.uwa.edu.au/auv/ftp/Underwater_datasets.zip). We used the BlueROV2 robot to acquire 1024 × 768 pixels images which are collected on an average of 12 frames per second. Images include part of the structure of the Remotely Operated Vehicle (ROV) (lights). Eight datasets are selected for the present chapter.

In Table 3, the selected datasets are described based on the underwater alterations explained in Section 3. The datasets covered sandy and rocky backgrounds with the presence of algae, far algae means that the algae is viewed as patches or are not moving, close algae means that algae is observed closely and movement is captured. Some datasets recorded isolated objects such as poles, rocks, part of a wreck and debris. The rotating over an object cell point out the frames involved in the navigation of the ROV around an object (frames in thousands).

### 4.4. Experimental Setup

We used a desktop computer with an Intel Core i7-7500U CPU @ 2.70 GHz × 4 CPU and 16 GB of RAM with Ubuntu 16.04 for the evaluation. The OpenCV [47] implementation of SIFT & SURF (non-free module xfeatures2d), AKAZE, ORB and BRISK are used. As well as the Nearest Neighbour (NN) algorithm for detecting matches between keypoints sets and Homography based on Random Sample Consensus (RANSAC) to reject outliers. The evaluation setup is based on the work found in [48] which integrates the OpenCV implementations in a friendly Graphical User Interface (GUI).

The modified program follows the block diagram presented in Figure 3 to perform our evaluation. The datsets are masked with a ‘Selected Area’ to exclude the lamps from the ROV which are easily recognizable by the detectors and appears in every frame causing inconsistencies in the matching process. The inliers and outliers are the feature matched between the two frames after computing the homography which ‘validates’ the correct matches (inliers) and false matches (outliers).

The data was logged into Comma Separated Values (CSV) files keeping the record of the number of features found, matches and processing time.

## 5. Results and Discussion

### 5.1. Detectable Features in Underwater Images

**Figure 4:**
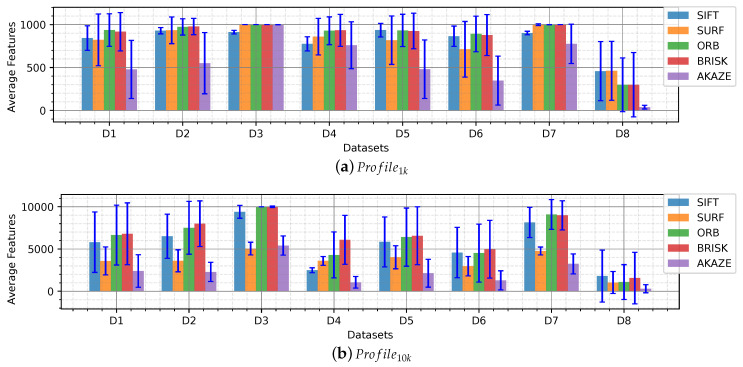
Detectable features in underwater images. Bar graph of the average number of features extracted per dataset with standard deviation (blue lines) given (**a**) $Profile_{1k}$ and (**b**) $Profile_{10k}$.

In Figure 4 an overview of the average features extracted per dataset is shown. The bar graphs show average values and the standard deviation to quantify the dispersion of the values obtained. The number of features indicates that the images present detectable salients which is the first step of a feature-based SLAM, to locate features. This number is used to describe in which underwater scenarios the detectors are more suitable and the performance of the detectors when compared to its pairs.

The overview shows an overall homogenous performance detecting around 500 features in $Profile_{1k}$ and 5000 features in $Profile_{10k}$ for all the detectors. Dataset_1 shows a high dispersion of the data for most of the detectors due to the sandy areas taken from far, similar to Datasets 4, 6 and 8, which present a low average. Conversely, Datasets 2, 3, 5 and 7, which present objects, rocks, algae patches, display a high average to the rest, being Datasets 3 and 7 the highest.

It is worth mentioning that the detailed graphs for both profiles have similar behavior, the only difference is the number which is proportional to the maximum number of features per profile. Therefore, in most cases, we analyze the $Profile_{1k}$ detail where the fluctuations, when finding a low number of features, are more evident than in $Profile_{10k}$.

**Figure 5:**
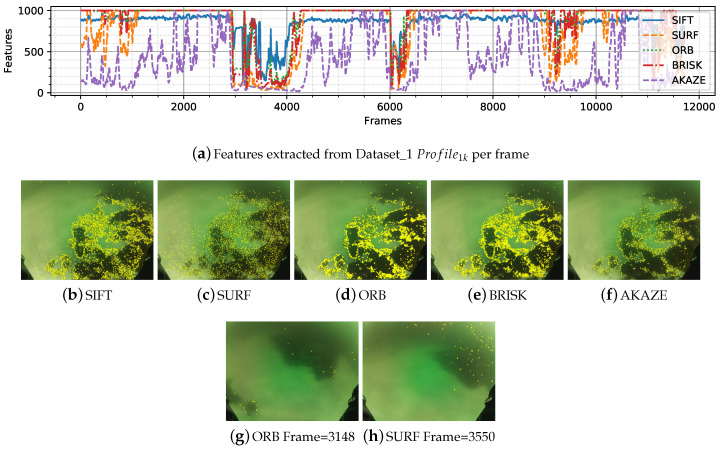
Detail of features extracted from Dataset_1. (**a**) features per frame. (**b**–**f**), features extracted with common feature extractors over the frame 710. (**g**,**h**) example of frames where the number of extracted features is low

We have selected two datasets to show the performance of the feature detectors in the underwater scenario. In Figure 5a can be seen the performance of the detectors applied to Dataset_1. Algae offer a good contrast on the sand exposing detectable features as seen in Figure 5b–f, it can be seen how ORB, BRISK, SIFT and AKAZE features surround the algae while SIFT features are more spare along the entire image. The figures also show that the detectors cannot find many features in plain sandy areas. During the frames ∼3000–∼4200 the ROV gets far from the seafloor, and the algae are seen as blurry patches, in this case, none of the detectors were able to extract much features (Figure 5g,h).

**Figure 6:**
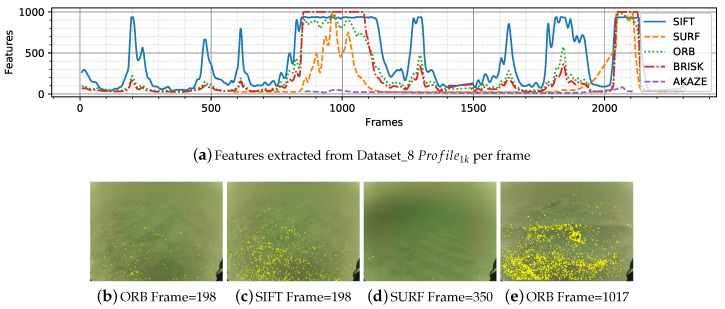
Detail of features extracted from Dataset_8. (**a**) features per frame. (**b**–**e**) extractors applied in different frames.

Dataset_8 is mostly sandy with some frames capturing partial poles as objects. The illumination is uniform and has a greenish tint (Table 3). As observed in Figure 5, plain sandy areas are hard environment to extract features from. Figure 6 shows the detail for Dataset_8. When the robot is close to the seafloor (20 cm approximately) the detectors start extracting features from the wavy pattern of the sand.

In overall, detectors are able to find features in underwater scenarios specially over rocks, defined algae patches (from far), objects and even sand (when exposing patterns). The detectors struggle in sandy areas from far as well as where turbidity and blurriness appears.

### 5.2. Frame Sequence Matching

**Figure 7:**
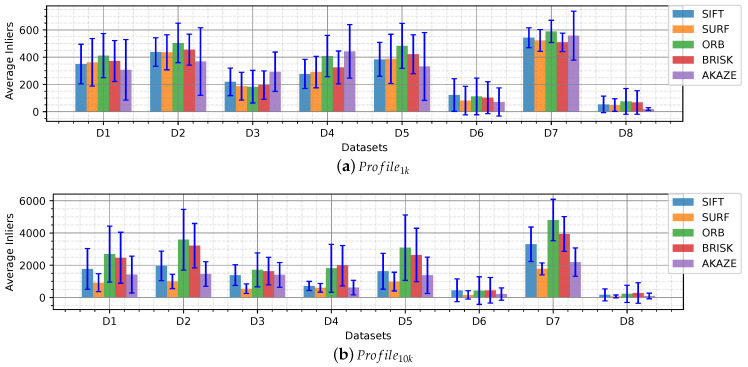
Inliers, obtained after NN matches and homography, per dataset.

It is important to quantify the number of features that can be re-observed (matched) in the following frames under the vSLAM scope. In this framework, the inliers (correct matches) are obtained after applying NN and homography between the keypoints detected in two consecutive frames. In Figure 7 a bar graph of the average inliers per dataset is shown. In this test, the descriptors obtained from the keypoints found with the detectors are evaluated.

Similar to the average number of features found, $Profile_{1k}$ and $Profile_{10k}$ show similar behavior for the different detectors. $Profile_{10k}$ show a lower number of inliers compared to its limit number of features (10,000) which means that a large number of features found in a frame is not matched in the consecutive frame.

Datasets 3, 4, 6 and 8 present the lowest average number of inliers, despite having a high average number of features found in Figure 4. Datasets 3, 4 and 6 have strong lighting issues such as caustic patterns and backscatter which are moving patterns that change rapidly between frames that are wrongly detected as features. Dataset 8, showed a low number of features found due to uniform texture displayed by sandy areas captured from far and got an even lower amount of inliers.

In the case of $Profile_{10k}$ SIFT, ORB and BRISK features slightly stick out compared to the others, especially in Datasets 2 and 7 which present defined algae areas from far and rocks, respectively.

AKAZE, which showed a lower number of average features extracted in Figure 4, shows around the same amount of inliers than the others which means that in this case the AKAZE detector was more robust than the others and only detected strong features that appeared in the consecutive frame, this can be easier to observe in the $recall_{m}od$ ratio analysis.

**Figure 8:**
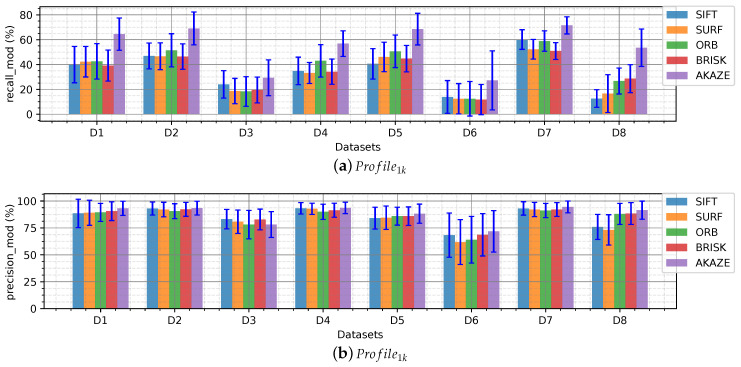
Inliers ratios per dataset.

Figure 8 shows the ratios presented in Equations (3) and (4), in percentage, for $Profile_{1k}$. In Figure 8a it can be seen that around 40% of the features found by the detectors are matched correctly in the consecutive frame. In other words, given 1000 features found in an frame, 400 features will be found and matched in the subsequent frame. AKAZE outstrips the other detector/descriptors in the performance, demonstrating that its extractor is more finicky than the others.

In Figure 8b can be observed that more than 75% of the features matched become inliers after homography indicating a good performance overall for the descriptors evaluated. That is to say that the descriptors are robust when describing keypoints in underwater scenarios.

### 5.3. Image Enhancement

**Figure 9:**
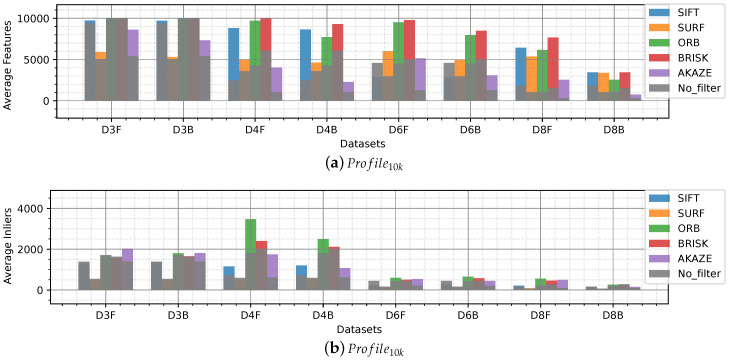
Results for pre-processed Datasets 3, 4, 6 and 8. Labels including an ‘F’ indicates pre-processed images applying the enhancement by fusion filter; labels including a ‘B’, back scatter removal filter.

We applied two image enhancement algorithms for underwater images to Datasets 3, 4, 6 and 8 which showed the lowest number of features or inliers found. In Figure 9 the enhancement by fusion filter [44] is represented by an ‘F’, and the backscatter removal filter [35], by a ‘B’. The results without any enhancement are shown in grey for easy comparison.

The average number of features extracted increases for Datasets 4, 6 and 8 (Figure 9a). Dataset_3, which is affected by light caustic patterns on a rocky background, does not show any improvement by any of the two algorithms. The image enhancement algorithm by fusion shows a better result exposing detectable features for the detectors.

It can be seen in Figure 9b that, in the case of SURF descriptors the number of features found presented and increase although, this increase is not observed at the time of matching those features in the consecutive frames (inliers). AKAZE benefits the most from the enhancement algorithms showing an improvement for all datasets. ORB, SIFT and BRISK are also helped by the algorithms in the order presented.

Dataset_4, which was taken at night with artificial illumination on a sandy background with few algae and rocks, gets the most significant improvement in the number of inliers. The filter by fusion gets better results than the backscatter filter.

Datasets 6 and 8 also increase their number of inliers, especially with the filtering by fusion. These two scenarios present a sandy background with few objects on the seafloor. Both present illumination problems, Dataset_6 presents a caustic pattern and Dataset_8 a non-uniform illumination.

### 5.4. Processing Time

**Figure 10:**
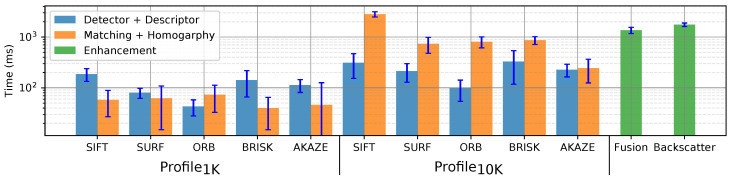
Processing average time per frame based on Dataset_2.

The processing time is measured for the detection and describing, NN matching and homography for the two profiles. In Figure 10, the average processing time for Dataset_2 is presented which also includes the pre-processing time for the enhancement algorithm.

ORB is the fastest set detector/descriptor with an average processing time of 43 ms and 97 ms for $Profile_{1k}$ and $Profile_{10k}$ respectively. SIFT and BRISK are the slowest with times around 150 ms and above 300 ms for $Profile_{1k}$ and $Profile_{10k}$. BRISK presents the highest dispersion having variations correlated with the number of features found, similar to SIFT; the rest show a continuous time for processing.

The enhancement algorithms applied are highly time-consuming showing values above 1 and 2 seconds for the filtering by fusion and backscatter removal algorithms respectively.

## 6. Conclusions

The experimental results provide a detailed analysis of SIFT, SURF, ORB, BRISK, and AKAZE detectors/descriptors for underwater environments towards their application to vSLAM.

In this analysis, the detectors selected showed a satisfactory performance on images containing color distortion, low non-uniform illumination and low turbidity. Sandy environments with algae patches, algae recorded from near and far; small particles, such as debris and rocks; and objects, such as poles and rocks presented detectable features for the detectors.

Different datasets were categorized according to the characteristics of the seafloor, types of objects, lighting, tint, and turbidity. The influence of these effects on the images is seen in the number of features extracted and subsequently matched in the following frames. The results showed a decrease of features and matches in presence of turbidity and blurriness, as in Figure 5a,g,f; monotony; sand patches with and without texture (Figure 6); and lighting, caustic patterns, shown in the overall number of features (Figure 4) and in the number of matches (Figure 7).

The number of inliers when matching keypoints from consecutive frames was homogeneous among the detectors, in $Profile_{10k}$ ORB and BRISK stick out. AKAZE achieved the best recall_mod ratio.

The two enhancement algorithm applied in this survey showed an improvement in the performance of the detectors/descriptors. The filter by fusion [44] showed the higher improvement especially in night scenarios with artificial light, caustic pattern and significant non-uniform illumination.

The survey provides abundant information and detailed insights valuable for making decisions in applications towards vSLAM. ORB detector/descriptor stood out in detection and matching performance, shaping up as a good selection for implementing vSLAM, with the lowest computing time.

## Figures and Tables

**Table 1:** Detectors/Descriptors characteristics and parameters.

Detector/Descriptor	Features to Detect	Size of Descriptor	Parameters
SIFT	Blobs	128 Bytes	Contrast Theshold, Sigma
SURF	Blobs	128 Float	Hessian Threshold
ORB	Corners	32 Bytes	Fast Threshold, Max Features
BRISK	Corners	64 Bytes	BRISK_threshold
AKAZE	Blobs	61 Bytes	AKAZE_threshold

**Table 2:** Profile parameters changes

	${\mathrm{Profile}}_{1k}$	${\mathrm{Profile}}_{10k}$
Max Features	1000	10,000
**Threshold**		
SIFT Contrast	0.01	0.008
SURF Hessian	60	8
ORB Edge	32	8
BRISK	10	7
AKAZE	0.0005	0.0001

**Table 3:** Datasets characteristics. The symbols >>, >, <, << are used to indicate the quantity: most, moderate, low and few, respectively

	Dataset_1	Dataset_2	Dataset_3	Dataset_4	Dataset_5	Dataset_6	Dataset_7	Dataset_8
**Seafloor**	sandy, algae (far), algae (close)	sandy, algae (far)	rocky, algae (far)	sandy, <algae (far)	sandy, algae (far)	sandy	sandy	sandy
**Objects**		poles		rocks	wreck	<debris	>>small rocks	<partial poles
**Light**	non-uniform	>>uniform	caustic pattern	night, backscatter	<<non-uniform	caustic pattern	>>uniform	>>uniform
**Tint**	greenish	greenish	natural	natural	greenish	natural	natural	greenish
**Turbidity**	low	low	low	low	low	low	low	low
**# Frames**	11729	5830	1929	8308	9155	2514	2522	2388
**Notes**		horizontal and vertical poles	little algae on rocks			robot shadow		wavy pattern on sand

## Abbreviations

The following abbreviations are used in this manuscript:

SLAM	Simultaneous Localization and Mapping
vSLAM	Visual SLAM

Feature Detectors and Extractors:

SLAM	Simultaneous Localization and Mapping
vSLAM	Visual SLAM
SIFT	Scale Invariant Feature Transform
SURF	Speeded-Up Robust Features
BRIEF	Binary Robust Independent Elementary Features
ORB	Oriented FAST and Rotated BRIEF
BRISK	Binary Robust Invariant Scalable Keypoints
AKAZE	Accelerated-KAZE
